# Sustainability reporting in the construction industry – Status quo and directions of future research

**DOI:** 10.1016/j.heliyon.2023.e21682

**Published:** 2023-11-02

**Authors:** Daniela Cortés, Albert Anton Traxler, Dorothea Greiling

**Affiliations:** Institute of Management Accounting, Johannes Kepler University, Altenberger Straße 69, 4040, Linz, Austria

**Keywords:** Construction industry, Corporate sustainability, Literature review, Sustainability reporting

## Abstract

The construction industry's activities have significant effects on nature, society, and economic development. These effects and the accompanied societal expectations have encouraged companies to deal with and report on their contributions to sustainable development (SD). While the state of the literature on sustainability reporting (SR) has already been mapped for other sectors, this has not yet happened for the construction industry. Through a systematic literature review, we identified 67 articles that examine companies' motivations for SR, the design and content of the reports, as well as the use of the information in corporate sustainability management. Literature predominantly suggests that sustainability disclosure is driven mainly by instrumental or social/political motives, and that reports vary greatly in terms of content and quality. The variations can be attributed to different factors. However, utilizing reporting to its full potential requires linking it with corporate strategy and adapting management practices accordingly. Although the review provides comprehensive insights, it also indicates further research needs.

## Highlights

• Research on the construction industry's sustainability reporting is at an early stage.
• Literature predominantly suggests instrumental or social/political motives for reporting.
• Studies show variations in reporting in terms of scope, quality and focus.
• Studies reveal ways how information can be used within sustainability management.
• There is a need for further research on this topic.

## 1 Introduction

In recent decades, companies in all sectors have expanded their reporting by publishing their ecological and social impacts in addition to the financial bottom line. Today, this form of reporting, commonly referred to as sustainability reporting (SR), is a de facto standard of corporate disclosure practice, especially for large or listed companies [[1]]. Given the significant influence companies belonging to the construction industry or their activities have on the physical and biological environment as well as on progress in social and economic matters [[2,3,4,5,6,7]], it is not surprising that they are now also increasingly disclosing their contribution to sustainable development (SD) in addition to their financial performance [e.g. Refs. [8,9,10]].

The construction industry includes all companies that carry out building and civil engineering activities and thus construct new buildings or civil engineering works, carry out renovations or modifications to existing ones, and erect prefabricated buildings or structures on the construction site [[11]]. Due to these activities, the industry is one of the major emitters of CO_2_ emissions directly through the construction, modification, and demolition of buildings and infrastructure, and indirectly since the energy demand in the operational phase of these buildings and infrastructures is also determined by their construction. The industry, therefore, plays a crucial role in making cities or communities more sustainable or providing sustainable infrastructure. However, the construction industry not only contributes to the ecological dimension of SD but also to social and economic matters, by helping to maintain or promote social cohesion and economic prosperity [e.g. Refs. [2,12,13,14]]. Disclosure that shows what contributions companies make to achieve SD, and thus gives an account of it, can help meet societal expectations and the information needs of stakeholders [[15,16,17,18]].

However, the motives for an increased focus on sustainability can be diverse for companies in the construction industry. For example, in addition to the desire to respond appropriately to growing societal expectations or increased stakeholder interests, they are also based on moral tenets of entrepreneurs and managers or strategic or instrumental advantages such as competitive advantages or advantages in terms of employee retention, to name just a few [[3,10,4]].

Companies that decide to report on their contribution to SD are generally not tied to a specific form of reporting when presenting their non-financial aspects. Nevertheless, sustainability reports have recently become more professional and precise due to the orientation toward reporting frameworks or guidelines, which guide the preparation of sustainability reports [[10]]. Well-known and widely used guidelines are the GRI (Global Reporting Initiative) standards, which cover a large number of non-financial topics and aim at transparent reporting [[19]]. In addition, legal requirements also determine the disclosure of non-financial information – for example, the European Union Directive 2014/95/EU [[20]] and the recently adopted Corporate Sustainability Reporting Directive (CSRD) [[21]] regulate the disclosure of non-financial and diversity information within the European Union.

However, it is important to mention that in addition to merely meeting legal requirements or external information demands, SR can also help companies implement sustainability measures and plan or control corporate sustainability (CS) performance and therefore significantly promote or enhance a company's contribution to SD [[15,22,23,17]].

Given the topic's relevance for the construction industry and the general trend towards the publication of sustainability reports, it is hardly surprising that a considerable number of publications have already covered the topic of SR in the construction industry. These studies examine SR from different perspectives: While some investigate the motives or drivers of reporting [e.g. Refs. [15,24,25,4]], others take a closer look at the content reported [e.g. Refs. [26,27,10]] or try to explain how the information gathered for reporting can be used to control CS performance [e.g. Refs. [28,29]].

However, many studies have already shown that industry affiliation can influence the distribution, content, or quality of the reports. In particular, the reporting of sectors with a significant impact on SD differs from those whose impact is comparatively lower [[15,30,31,17]]. Hence, it makes sense to present the evidence on reporting in a sector-wise overview. The empirical evidence regarding SR has been mapped and synthesized via literature reviews in other sectors with significant impacts on SD, including mining [[32]] or the airline industry [[33]]. The construction industry, however, lacks such an overview despite its numerous characteristics, which suggest differences in reporting behavior and report content, severely limiting the transferability of findings from other industries. The GRI also recognized these industry specifics in terms of reporting at an early stage and took them into account by publishing a sector supplement for the construction and real estate industry in 2011, updated in 2013, and currently developing new standards which also addresses the specific industry requirements [[34]].

Against this background, our literature review aims to present the current state of literature on SR in the construction industry, to synthesize the findings and to outline possible avenues for future research. In doing so, we aim to not only answer which content is reported on how comprehensively and what influences this but also what motivations and drivers cause companies in the construction industry to report on sustainability at all. Furthermore, it is also intended to show what we know about the role of reporting within CS management or how the companies use the information gathered.

To accomplish this and to structure our investigation, we asked the following research questions.

RQ 1 Why are companies in the construction industry engaged in sustainability reporting?

RQ 2 What do the design and content of sustainability reports of companies from the construction industry look like?

RQ 3 How is sustainability reporting used within the corporate sustainability management of companies in the construction industry?

The paper is structured as follows. Section 2 – **Theoretical discourse on sustainability reporting in the construction industry** – outlines theoretical perspectives that not only help to explain the motives for reporting but might also explain variations in the design or content of the reports as well as the different uses of the information gathered for CS management. The methodological approach applied in this study, is presented in Section 3 –**Systematic literature review**. Section 4 – **Results** – presents the findings of our study. In Section 5 – **Discussion of results** – these are discussed and directions for further research are outlined before Section 6 – **Conclusion, limitations & implications** draws pertinent conclusions.

## 2 Theoretical discourse on sustainability reporting in the construction industry

SR aims to present the company's measures, developments and progress in the area of sustainability transparently [e.g. Refs. [35,36]]. In line with this, Schaltegger [[37]] defines it as *“[…] the formal and official corporate communication which provides information about corporate sustainability issues. This includes in particular information about the social, environmental and economic performance and the relationships between these aspects of corporate performance.”* [[37], p. 184] The GRI also emphasizes the creation of transparency and accountability in the presentation of the objectives of its reporting framework, as follows: *„The objective of the GRI Sustainability Reporting Standards (GRI Standards) is to provide transparency on how an organization contributes or aims to contribute to sustainable development. The GRI Standards enable an organization to publicly disclose its most significant impacts on the economy, environment, and people, including impacts on their human rights and how the organization manages these impacts. This enhances transparency on the organization's impacts and increases organizational accountability.”* [[19], p. 7]. However, in addition to creating transparency or accountability, SR can also support companies in implementing sustainability measures or activities or managing CS performance [[15,16,22,23,17,18]]. Therefore, it has the potential to support or enhance CS management and noticeably enhance a company's contribution to SD [ [38]].

The proportion of companies disclosing information on their contributions to SD has increased not only in general but also in the construction industry [[1]]. As to why a growing number of companies in the construction industry are engaging in reporting, the literature indicates a wide range of reasons. Foremost among these are the awareness of one's environmental impact, such as land consumption or CO_2_ emissions in all phases such as the material manufacturing, construction, use, maintenance and demolition phases [[39]], the associated legitimacy needs [e.g. Refs. [3,4,40]], the fulfillment of legal requirements [e.g. Ref. [41]], the attainment of competitive advantages [e.g. Ref. [42]], the integration of sustainability measures to make corporate actions more sustainable [e.g. Ref. [22]], or the provision of information for different stakeholders [e.g. Ref. [24]].

However, the various reasons for companies to devote themselves to the topic of sustainability, establish appropriate control practices in this context, or begin with SR can be assigned to three different theoretical strands – the instrumental, the social/political, and the normative perspective – each with different rationales or drivers behind them. Companies that start reporting beyond the financial bottom line for instrumental or strategic reasons expect the reporting activity to contribute to the achievement of traditional corporate goals, such as improving access to the capital market or increasing market shares [[43]]. Several theories can be assigned to this instrumental view. The agency theory and the strategic branch of the stakeholder theory [[44,45]] are well-known examples that are often used as explanatory approaches in the context of SR by companies [e.g. Refs. [26,46,25]].

From these perspectives, the sustainability report serves as an instrument to reduce existing information asymmetries between the company and the strategically relevant stakeholders regarding the CS performance [[47,48,49,50]]. The construction industry is characterized by a particularly complex stakeholder structure, as construction projects involve a large number of different stakeholders, such as customers, government agencies, contractors and sub-contractors, or planners and architects, to name just a few [[47,50]]. These stakeholders can have different levels of power and can exert a significant influence on a company. However, sustainability reports can help to respond appropriately to stakeholders’ interests and information needs [[51,50,52]]. Thereby, from an instrumental perspective, an expansion of the shareholder focus in corporate reporting makes sense, since the inclusion of other strategically relevant stakeholders the company depends on ensures the continued success of the company [[53]].

In contrast, the social/political perspective assumes that companies in the construction industry do not value SR because of its instrumental value, but rather as a tried and tested means of legitimizing corporate activities or, in other words, as an instrument to react to societal expectations to gain, maintain or defend organizational legitimacy [[43,4]]. In this vein, the prevailing societal expectations drive or shape companies’ SR [[43,25,54]]. The pressure to legitimize or justify business activities is particularly high for companies that belong to sectors that are subject to great public awareness or scrutiny [[55,4]]. Given the impact of the sector on the environment, society, and economic development [e.g. Refs. [2,12,13,14]], this is true in the construction industry to a comparatively high degree.

Legitimacy, however, is not solely understood by companies as a strategic resource [[56]] but rather considerations or requirements of legitimacy also result from institutional pressure within a field [[57]]. This pressure to legitimize corporations' actions or, subsequently, to publish social and ecological information can differ substantially and ranges from formal compliance requirements in the form of laws, like the European Union Directive 2014/95/EU [[20]] and the CSRD [[21]], to non-codified expectations of specific institutional actors or stakeholders [[58,59]]. The pressure ultimately leads to homogenization within a field and to an adjustment of reporting behavior by companies within it [[60,57]].

Against both the instrumental and the social/political perspective, companies whose motive for SR can be assigned to the normative view report neither to achieve strategic goals nor for reasons of legitimacy, but see it as a moral obligation to provide all stakeholders of the company with information regarding its contribution to SD. Accordingly, the publication of additional information is not a question of whether it is of interest to the strategically relevant stakeholders and whether advantages can therefore be expected from addressing it; rather, the interests of all stakeholders are taken into account to comply with the company's perceived moral obligation [[44,43,61,62]].

## 3 Systematic literature review

Our study covers the currently available literature with the purpose of mapping and synthesizing the existing literature in the field and outlining potential avenues for further research [[63,64,65]]. This research follows the guidance of the Preferred Reporting Items for Systematic Reviews and Meta-Analyses (PRISMA), which is a 27-item reporting guideline to ensure a complete, comprehensible, and transparent process and account of a systematic literature review [[64]]. First, research questions were developed, followed by a selection of the databases for the literature search; then, the search terms were chosen and search strings developed, followed by a practical screening and a methodological screening, the execution of the review itself, and finally a synthesis and discussion of the results.

The keywords for our literature search were derived from an initial intensive literature study and then divided into two sub-areas. The first section deals with SR and its synonyms, such as CSR reporting, TBL reporting, or integrated reporting, while the second section focuses on the industry reference. Due to the early stage of the literature, the often-broad spectrum of activities of companies in the construction industry along the entire value chain in the areas of construction and real estate, and the lack of concrete industry definitions in several studies, we decided to broaden the keywords in terms of industry restriction to be able to identify as many findings as possible that are relevant to our topic. The following Table 1 shows the search string.

**Table 1:** Search String^1^.

(“sustainability!report*” OR “sustainability!disclosure*" OR “corporate!social!responsibility!report*" OR “CSR!report*" OR “triple!bottom!line!report*" OR “TBL!report*" OR “integrated!report*" OR “global!reporting!initiative” OR “GRI” OR “international!integrated!reporting!council” OR “IIRC”)	AND	(“building!construct*" OR “building!industr*" OR “building!sector” OR “property!sector” OR “property!industr*" OR “construction!industr*" OR “construction!sector” OR “construction!field*" OR “construction!compan*" OR “construction!work” OR “sustainable!construct*" OR “sustainable!building*" OR “green!building*" OR “green!construct*")

The search for articles was carried out in the databases EbscoHost, Science Direct, Scopus, and Web of Science. In each of these databases, the inclusion criterion was that identified keywords of the search string must appear in the title, abstract, or keywords. In addition, the results were filtered for publications in academic journals up to the end of 2022, with only journal articles included. Other publications such as book reviews, or editorial notes were excluded.

Due to the aim of presenting the state of the literature in its entirety and the manageable number of available publications on this topic, no quality restrictions (e.g., by using certain rating requirements or impact factors) were imposed. Using these inclusion and exclusion criteria, all articles that were found were checked for their content, i.e., whether they related to SR in the construction industry or not.

In the first step, the initial total of 311 articles was checked for duplicates which then were removed (resulting in 293 articles) and the title and abstract were checked for content. In case of uncertainties regarding the content fit for the review, the authors exchanged information with each other to prevent the risk of biased assessment. Title and abstract analyses that showed a misfit for the review purpose led to the exclusion of the respective articles (resulting in 107 articles). The remaining articles were subjected to deep full-text analysis and, if necessary, eliminated in case of discrepancies due to the focus of the content (for example, due to a strong technical orientation of the articles). Here, too, the author team coordinated with each other to guarantee a reliable selection of articles.

**Fig. 1:**
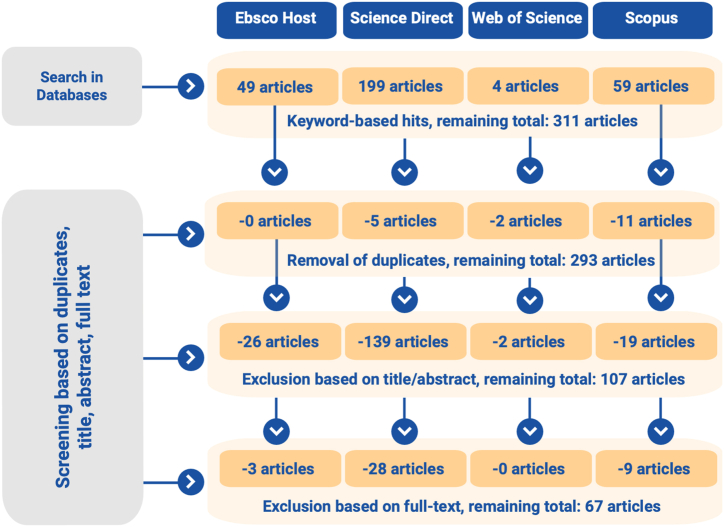
Prisma flowchart.

Fig. 1 shows the process of identification and selection in the different steps using a PRISMA flowchart [[64]].

The contents of the 67 selected articles were subsequently analyzed in detail to determine the current state of the literature on SR in the construction industry. The identified articles investigated the topic primarily with mixed approaches that work quantitatively and qualitatively by analyzing the content of sustainability reports, while some articles used exclusively qualitative research designs with interviews and/or case studies in companies from the construction industry, or exclusively quantitative research designs with surveys, or approached the topic conceptually. The following Table 2 provides an overview of the identified articles along with these findings.

**Table 2:** Article sample.

No.	Title	Author(s)	Year	Journal	Method	Theory
1	Stakeholder engagement: A green business model indicator	Abuzeinab & Arif	2014	Procedia Engineering	qualitative	N/A
2	An investigation of corporate approaches to sustainability in the construction industry	Afzal et al.	2017	Procedia Engineering	mixed approach	stakeholder theory
3	Is the social agenda driving sustainable property development in Melbourne, Australia?	Ang & Wilkinson	2008	Property Management	quantitative	N/A
4	Sustainability in the Brazilian Heavy Construction Industry: An Analysis of Organizational Practices	Arruda et al.	2013	Sustainability	mixed approach	N/A
5	Malaysian construction firms' social sustainability via organizational innovativeness and government support: The mediating role of market culture	Bamgbade, Kamaruddeen & Nawi	2017	Journal of Cleaner Production	conceptual	resource-based view
6	UK and Italian EIA systems: A comparative study on management practice and performance in the construction industry	Bassi et al.	2012	Environmental Impact Assessment Review	mixed approach	N/A
7	Creating value by sustainable manufacturing and supply chain management practices – a cross-country comparison	Campos et al.	2017	14th Global Conference on Sustainable Manufacturing	mixed approach	N/A
8	Facilitating the transition to sustainable construction: China's policies	Chang et al.	2016	Journal of Cleaner Production	mixed approach	N/A
9	Sustainability attitude and performance of construction enterprises: A China study	Chang et al.	2018	Journal of Cleaner Production	quantitative	stakeholder theory
10	Relationships between Environmental Initiatives and Impact Reductions for Construction Companies	Chang, Canelas & Chen	2021	Sustainability	mixed approach	N/A
11	Linking key topics to environmental indicators in corporate social responsibility reports of construction companies	Chang, Paramosa & Tsai	2021	Corporate Social Responsibility and Environmental Management	mixed approach	stakeholder theory
12	Environmental indicator disclosure of international contractors	Chang, Romero & Tsai	2022	Journal of the Chinese Institute of Engineers	mixed approach	N/A
13	The linkages between internationalization and environmental strategies of multinational construction firms	Chen, Ong & Hsu	2016a	Journal of Cleaner Production	mixed approach	resource-based view
14	Understanding the relationships between environmental management practices and financial performances of multinational construction firms	Chen, Ong & Hsu	2016b	Journal of Cleaner Production	mixed approach	standard microeconomic theory, signaling theory, resource-based view
15	Occupational health and safety disclosures in sustainability reports: An overview of trends among corporate leaders	Evangelinos et al.	2018	Corporate Social Responsibility and Environmental Management	mixed approach	N/A
16	Exploring the status of corporate social responsibility disclosure in the UK building and construction industry	Evangelinos et al.	2016	International Journal of Global Environmental Issues	mixed approach	legitimacy theory, stakeholder theory, accountability theory
17	The state of sustainability reporting in the construction sector	Glass	2012	Smart and Sustainable Built Environment	mixed approach	N/A
18	Sustainability integration in the management of construction projects: A morphological analysis of over two decades' research literature	Goel, Ganesh & Kaur	2019	Journal of Cleaner Production	conceptual	stakeholder theory
19	Evolving green building: triple bottom line or regenerative design?	Gou & Xie	2017	Journal of Cleaner Production	conceptual	N/A
20	Sentiment analysis trend on sustainability reporting in Indonesia: evidence from construction industry	Harymawan et al.	2020	Journal of Security and Sustainability Issues	mixed approach	N/A
21	Understanding stakeholders' approaches to sustainability in building projects	Herazo & Lizarralde	2016	Sustainable Cities and Society	mixed approach	stakeholder theory
22	The current conditions of CSR implementation in construction industry: a lesson from Taiwan	Huang et al.	2017	Applied Ecology and Environmental Research	mixed approach	N/A
23	Key credit criteria among international green building rating tools	Illankoon et al.	2017	Journal of Cleaner Production	mixed approach	N/A
24	Understanding building sustainability – the case of Sweden	Isaksson & Rosvall	2020	Total Quality Management	mixed approach	process theory
25	What does GRI-reporting tell us about corporate sustainability?	Isaksson & Steimle	2009	The TQM Journal	mixed approach	stakeholder theory, systems theory, The Natural Step theory
26	Key activity areas of corporate social responsibility (CSR) in the construction industry: a study of China	Jiang & Wong	2016	Journal of Cleaner Production	mixed approach	N/A
27	Corporate social responsibility and the UK construction industry	Jones, Comfort & Hillier	2006	Journal of Corporate Real Estate	mixed approach	N/A
28	Materiality and external assurance in corporate sustainability reporting - An exploratory of Europe's leading commercial property companies	Jones, Hillier & Comfort	2016	Journal of European Real Estate Research	mixed approach	stakeholder theory
29	Commercial property investment companies and corporate social responsibility	Jones et al.	2009	Journal of Property Investment & Finance	mixed approach	N/A
30	Lean and sustainable construction: link between the sustainability report disclosure and the impact on profitable opportunities for investors	Kresnanto & Putri	2019	Materials Science and Engineering	mixed approach	N/A
31	Exploring the nexus between integrated reporting and sustainability embeddedness	Le Roux & Pretorius	2019	Sustainability Accounting, Management and Policy Journal	qualitative	sensemaking theory
32	Communicating the corporate social responsibility (CSR) of international contractors: content analysis of CSR reports	Liao et al.	2017	Journal of Cleaner Production	mixed approach	stakeholder theory
33	Does corporate social performance pay back quickly? A longitudinal content analysis on international contractors	Liao et al.	2018a	Journal of Cleaner Production	mixed approach	stakeholder theory, moral philosophy theory, neoclassical economic theory, good management theory, private cost theory
34	Comparing international contractors' CSR communication patterns: A semantic analysis	Liao et al.	2018b	Journal of Cleaner Production	mixed approach	agenda-building theory, stakeholder theory
35	How socially responsible is construction business in Australia and New Zealand?	Lim & Loosemore	2017	Procedia Engineering	quantitative	N/A
36	Stakeholders' influence strategies on social responsibility implementation in construction projects	Lin et al.	2019	Journal of Cleaner Production	qualitative	resource dependence theory
37	Who should take the responsibility? Stakeholders' power over social responsibility issues in construction projects	Lin, Ho & Shen	2017	Journal of Cleaner Production	quantitative	social contract theory, resource dependence theory, stakeholder network theory, multiplicity theory
38	A comparison of corporate social responsibility practices in the Singapore, Australia and New Zealand construction industries	Loosemore et al.	2018	Journal of Cleaner Production	quantitative	institutional theory
39	Corporate social responsibility disclosures in international construction business: trends and prospects	Lu et al.	2016	Journal of Construction Engineering and Management	mixed approach	legitimacy theory, stakeholder theory
40	Corporate sustainability for architecture engineering and construction (AEC) organizations: Framework, transition and implication strategies	Lu & Zhang	2016	Ecological Indicators	mixed approach	N/A
41	Corporate social responsibility disclosure in China: Do managerial professional connections and social attention matter?	Luo & Liu	2020	Emerging Markets Review	mixed approach	agency theory, legitimacy theory, stakeholder theory, resource-based view, prospect theory
42	The Boundaries of Reporting Sustainable Development in Social Housing	Manochin, Jack & Howell	2008	Public Money & Management	qualitative	N/A
43	Occupational health and safety of multinational construction companies through evaluation of corporate social responsibility reports	Mavroulidis et al.	2022	Journal of Safety Research	mixed approach	stakeholder theory
44	Environmental and Social Sustainability in UK Construction Industry: a Systematic Literature Review	Misopoulos	2019	European Journal of Economics and Business Studies	conceptual	institutional theory
45	Corporate responsibility reporting in UK construction	Moon, Parry & Brown	2009	Engineering Sustainability	mixed approach	N/A
46	Leveraging effects of triple bottom lines business model on the building and construction small and medium-sized enterprises' market performance	Okanga & Groenewald	2017	Acta Commercii - Independent Research Journal in the Management Sciences	qualitative	N/A
47	Implementation of the Global Reporting Initiative Social Sustainability Indicators	Passos Neto et al.	2022	Sustainability	conceptual	N/A
48	Sustainability Information in Annual Reports of Companies Domiciled in the Czech Republic and the Slovak Republic	Petera et al.	2019	Inzinerine Ekonomika-Engineering Economics	mixed approach	shareholder theory, stakeholder theory, legitimacy theory, institutional theory
49	The disclosure of anticorruption aspects in companies of the construction sector: Main companies worldwide and in Latin America	Saenz & Brown	2018	Journal of Cleaner Production	mixed approach	N/A
50	Critical Evaluation of Environmental, Social and Governance Disclosures of Malaysian Property and Construction Companies	Siew	2017	Construction Economics and Building	mixed approach	N/A
51	Sustainable construction practice and contractors' competitiveness: A preliminary study	Tan, Shen & Yao	2011	Habitat International	conceptual	N/A
52	A study of sustainable practices in the sustainability leadership of international contractors	Tan et al.	2020	Sustainable Development	mixed approach	N/A
53	Combining process analysis method and four-pronged approach to integrate corporate sustainability metrics for assessing international construction joint ventures performance	Tetteh, Chan & Nani	2019	Journal of Cleaner Production	conceptual	N/A
54	The curvilinear relationship between corporate social performance and corporate financial performance: Evidence from the international construction industry	Wang et al.	2016	Journal of Cleaner Production	qualitative	transaction cost theory, stakeholder theory, resource dependence theory, legitimacy theory
55	Balanced sustainable implementation in the construction industry: The perspective of Korean contractors	Whang & Kim	2015	Energy and Buildings	mixed approach	N/A
56	Conceptualising the state of the art of corporate social responsibility (CSR) in the construction industry and its nexus to sustainable development	Xia et al.	2018	Journal of Cleaner Production	mixed approach	social contract theory
57	Understanding the CSR Awareness of Large Construction Enterprises in China	Xie et al.	2020	Advances in Civil Engineering	mixed approach	stakeholder theory
58	A framework model for assessing sustainability impacts of urban development	Xing et al.	2009	Accounting Forum	mixed approach	N/A
59	Virtuous nexus between corporate social performance and financial performance: a study of construction enterprises in China	Xiong et al.	2016	Journal of Cleaner Production	mixed approach	stakeholder theory, slack resource theory, good management theory
60	Corporate social responsibility “glocalisation”: Evidence from the international construction business	Ye et al.	2020	Corporate Social Responsibility and Environmental Management	mixed approach	N/A
61	Impact of Institutional Distance on Environmental and Social Practices in Host Countries: Evidence from International Construction Companies	Ye, Lu & Xue	2022	Journal of Construction Engineering and Management	mixed approach	institutional theory
62	An evaluation of sustainable construction perceptions and practices in Singapore	Yin et al.	2018	Sustainable Cities and Society	quantitative	N/A
63	Drivers, motivations, and barriers to the implementation of corporate social responsibility practices by construction enterprises: a review	Zhang, Oo & Lim	2019	Journal of Cleaner Production	conceptual	organizational learning theory, institutional theory, stakeholder theory, self-determination theory
64	Corporate social responsibility practices by leading construction firms in China: a case study	Zhang, Oo & Lim	2022	International Journal of Construction Management	mixed approach	stakeholder theory, institutional theory, self-determination theory, sustainability transition theory
65	A corporate social responsibility indicator system for construction enterprises	Zhao et al.	2012	Journal of Cleaner Production	mixed approach	stakeholder theory
66	Sustainability policy of construction contractors: A review	Zuo et al.	2012	Renewable and Sustainable Energy Reviews	mixed approach	N/A
67	Green building research–current status and future agenda: A review	Zuo & Zhao	2014	Renewable and Sustainable Energy Reviews	conceptual	stakeholder theory

## 4 Results

### 4.1 Descriptive analysis

In the following, descriptive analyses of the 67 identified articles regarding publication medium, publication years, and geographical targeting are presented.

#### 4.1.1 Publications by journal

**Fig. 2:**
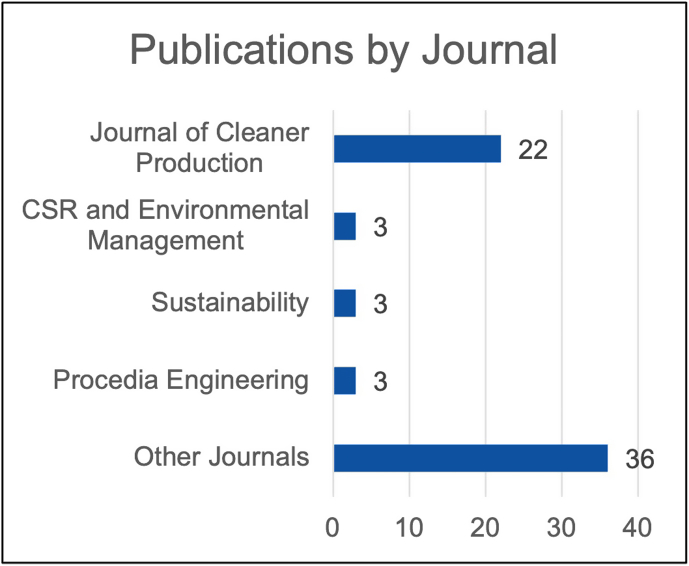
Publications by journal.

Fig. 2 shows that publications on the topic of SR in the construction industry are spread across a relatively large number of journals, with around a third of the articles being published in the “Journal of Cleaner Production”. Three articles each were published in the journal “Corporate Social Responsibility and Environmental Management”, “Procedia Engineering” and “Sustainability”, while the other articles were published in a variety of journals.

#### 4.1.2 Publications per year

As mentioned above, all articles up to the end of 2022 were included. With the presence of the draft of the first version of the GRI guidelines and the introduction of the Dow Jones Sustainability Index in 1999, SR has gradually gained attention from around the year 2000. Therefore, earlier contributions are rather unlikely.

**Fig. 3:**
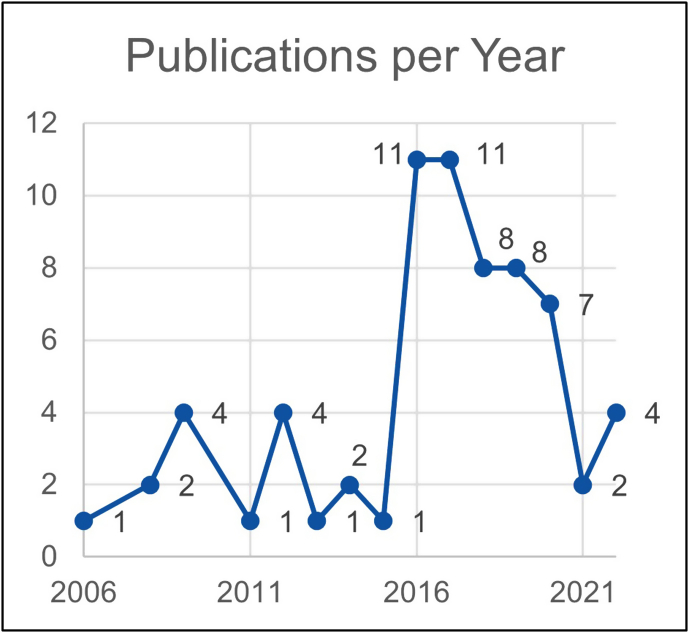
Publications per year.

Fig. 3 shows that the first article was published in 2006 and dealt with the CSR of construction companies in the UK. In 2016 and 2017, the number of publications over the years peaked with eleven published articles each year.

#### 4.1.3 Publications by geographical focus

**Fig. 4:**
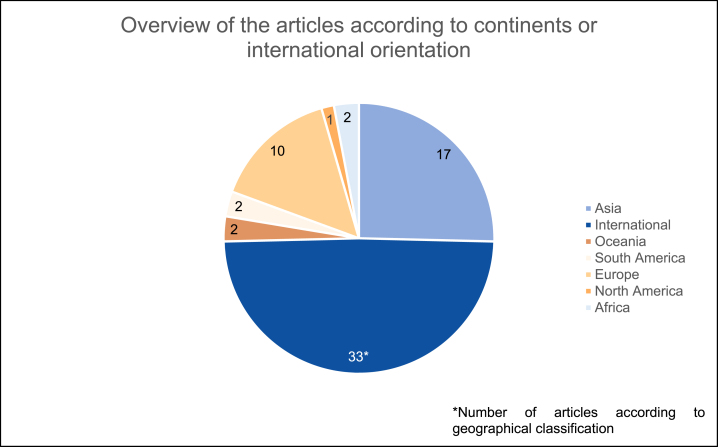
Geographical classification.

The publications were checked for their geographical orientation (e.g., a study is examining sustainability reports from companies belonging to the construction industry in a specific country) and classified according to continents or geographical regions. Of a total of 67 articles, 33 publications – i.e., approximately half – dealt with SR in the construction industry on an international level. More than a quarter of the contributions (17 articles) focused on the Asian area whereas ten articles dealt with the topic by considering European countries. Two articles dealt with sustainability reports of the construction industry in Brazil and were therefore appointed to South America, and another one deals with stakeholder approaches to building projects in Canada and is therefore assigned to the North American continent. Additionally, a total of four publications come from Africa and Oceania. Fig. 4 shows the geographical distribution of the samples in the analyzed articles.

### 4.2 Content evaluation

After a descriptive analysis of the 67 identified contributions, these were examined regarding their content. Subsequently, the papers were assigned to thematic clusters differentiated in terms of motives and drivers of SR, contents and designs of SR, and SR and corporate sustainability management. However, multiple assignments were also possible, depending on the thematic focus or foci of the individual articles. Table 3 provides an overview of these clusters.

**Table 3:** Thematic orientation of articles by cluster.

Cluster	Assigned Articles	Number of articles
Motives and drivers of sustainability reporting	3, 4, 5, 7, 8, 9, 14, 15, 16, 17, 19, 20, 22, 26, 27, 28, 29, 30, 31, 33, 34, 36, 38, 41, 45, 48, 51, 52, 54, 55, 56, 57, 59, 60, 61, 62, 63, 64, 66	39
Contents and designs of sustainability reports	2, 3, 4, 9, 11, 12, 13, 14, 15, 16, 17, 20, 22, 23, 24, 25, 26, 27, 28, 29, 32, 33, 34, 35, 38, 39, 40, 43, 44, 45, 46, 47, 48, 49, 50, 52, 55, 57, 60, 61, 64, 66, 67	43
Sustainability reporting and corporate sustainability management	1, 2, 3, 4, 5, 6, 7, 9, 10, 12, 13, 17, 18, 21, 23, 24, 28, 29, 31, 32, 34, 35, 36, 37, 38, 39, 42, 43, 46, 51, 53, 54, 55, 58, 62 63, 65, 66, 67	39

Chapter 4.2.1. with all its subchapters is dedicated to answer research question 1, which asks why companies in the construction industry engage in SR.

#### 4.2.1 Motives and drivers of sustainability reporting

##### 4.2.1.1 Instrumental approach

Reasons for addressing SR and/or implementing sustainability activities in a company can be based on rational instrumental or strategic considerations. The effects of the construction industry's actions draw attention from society and have led companies to publicly and transparently present information on their contributions to SD [[3,4]]. A study of Chinese contractors, for example, showed that strategically assessing the potential loss or gain in value through socially responsible or irresponsible behavior can impact CS performance and reporting on it [[25]]. The influence of strategic or instrumental considerations on reporting can also be seen in the supply chain. Considering the supply chain of construction companies, long-term business partnerships and collaborations can be effective drivers for the proactive implementation of SR in the whole industry [[66]].

In addition, the competitive pressure in the industry also plays a decisive role. A study of Brazilian companies in the construction industry showed that the motivation for sustainability and related reporting is shaped by a highly competitive market environment with changing production standards, globalization, specific demands, and environmental regulations [[67]]. Moreover, business growth, a planned stock listing or listing on the OTC market, or a planned expansion into other continents/oversea regions may constitute motives for SR [[68]]. Frameworks, regulations, and standards could also help improve CS performance, reporting, and related competitiveness [[28,42,69]].

There are often overlaps between the ecological or social and financial performance of a company, which means that considering ecological and social measures also makes sense from a financial perspective. SR itself can help identify and better understand the connections or correlations between the dimensions [[22]]. Whang and Kim [[14]] found, e.g., a strong correlation between ecological and economic factors. Jones et al. [[70]] mention that CSR commitments must be balanced with commercial considerations, in order to avoid internal tensions. Some authors [[71,72,73,74,40,59]] emphasize that considering and accounting for global CSR issues in the long term could help internationally operating companies to generate potential cost savings and thus translate into financial outcomes, and ensure continuity and efficient operations while harmonizing CSR practices.

Evangelinos et al. [[15]] took a closer look at the health and safety reporting of international construction companies and found possible connections between reporting, and the resulting improved monitoring of health and safety risks and enhanced communication with stakeholders. Another strategic or instrumental benefit, and thus a driver that can accrue from reporting, is an increase in the number of customers who value companies that report information on, for example, employee working conditions [[40,18]].

##### 4.2.1.2 Social/political approach

In addition to instrumental or strategic factors, social/political aspects also represent reasons for increased engagement with SR. The environment of a company or the framework conditions and the associated societal expectations can drive companies to engage in sustainability activities to gain or maintain legitimacy, as companies attempt to respond to these framework conditions or expectations by means of CSR measures, in particular also by referring to local and contextual conditions [[75,42,73,76,59,77,18]]. The idea of legitimacy is also considered by Petera et al. [[4]], who explain that companies belonging to an industry that is under strict public scrutiny must justify their behavior, for which sustainability reports can be used. From the point of view of Liao et al. [[78]], it is important that managers spend more time, money, and work on meeting the increased information needs of stakeholders. Due to the large number of stakeholders involved in construction projects, it is particularly important to show that responsibility (e.g., through the transparent presentation of decision-making processes) is assumed towards all stakeholders [[3]]. Evangelinos et al. [[8]] describe the reported CSR information as a necessary fulfillment of social accountability and crucial to improving communication with stakeholders. The information should help stakeholders analyze the economic, environmental, and social decisions of companies and increase companies’ accountability [[24]].

However, the actual measures taken by a company in the area of sustainability must be consistent with the information published about them. Many authors [[15,79,22,80,14]] focus on a comparison between the actual performances of sustainability activities and the importance given to sustainability or its reporting. Occasionally, aspects of so-called “greenwashing” are mentioned in the literature, i.e., suspicions that companies in practice do not comply with the measures reported in their sustainability report. Some authors [[67,[70], [81], [82],^80^,^14^]] indicate a large gap between the communicated relevance of sustainability aspects and actual performance. Gou and Xie [[79]] even speak of a rhetorical generation that deals with the definition of concepts, frameworks, indicators, and rating systems instead of taking physical measures. Evangelinos et al. [[8]] go even further and speak of artificial information provision as well as mere window dressing. In contrast, Campos et al. [[83]] critically mention that one should not equate potential gaps in sustainability reports or underreported topics with a lack of consideration or activities in specific areas. Le Roux and Pretorius [[22]], on the other hand, found a broad correspondence between communicated measures for more sustainability and the actual performance of companies, speaking of a cogwheel system between the embedding of sustainability and integrated reporting.

In summary, it can be said that the reasons and drivers for becoming more involved with SR are very diverse. On the one hand, these can be of an instrumental or strategic nature, if motives such as an improvement in competitiveness, expansion of business activities, business growth, long-term partnerships, and collaborations, cost savings, more efficient project handling, improved communication with strategically relevant stakeholders or expansion of the customer base are in the foreground. On the other hand, social/political considerations can also be drivers, for example, in aligning sustainability measures with stakeholder expectations, addressing the requirements of a particular region, or social accounting to foster relationships with a company's stakeholders. However, in order to gain the trust of stakeholders, it is important to ensure that the communicated sustainability measures also correspond to the activities actually undertaken by the company. Certainly, normative motivations (e.g., the moral attitude towards sustainability of CEOs) can also contribute to intensive engagement with SD and associated reporting. However, this is not or only marginally addressed in the literature on SR in the construction industry [e.g. Ref. [25]].

The following chapter 4.2.2. with all subchapters answers research question 2, which refers to the contents and designs of sustainability reports.

#### 4.2.2 Contents and designs of sustainability reports

##### 4.2.2.1 Variations in reporting

The nature and scope of sustainability reports in the construction industry vary widely. As early as 2009, Jones et al. [[70]] recognized that different priorities are set on individual dimensions of sustainability or that individual measures are particularly emphasized. When looking at sustainability reports from international construction companies, depending on the geographical location, differences and variations in the design of the reports concerning specific dimensions of sustainability and specific foci on topics were found [[2,81,51]]. For example, European and US construction companies had better CSR communication in an international comparison than, for example, Chinese ones, concerning working practices and conditions [[78,51]]. The study by Lu et al. [[10]] also found a regional influence on an overall level. Their findings suggest that economically highly developed countries have a higher level of CSR reporting than economically less developed countries.

Besides country-specific circumstances, other factors, such as internationalization and size, also influence reporting practices. For example, it was shown that larger companies report more comprehensively [[27,68,4,59,77,84]]. Furthermore, effects of the accounting standards used for reporting were also identified [[4]].

Variations in SR have also been identified along the value chain. Moon et al. [[85]] recognized, for example, that the UK construction industry materials suppliers align their reporting more closely to the key performance indicators of the GRI guidelines than other participating business sectors in the UK construction industry (e.g., consulting firms). Jones et al. [[82]] criticize that a common focus on essential content and sector-specific approaches to materiality as part of the SR processes is missing. Xie et al. [[17]] as well as Lim and Loosemore [[86]] state that there are also differences in the perception, the resulting derivation of sustainability activities, and the representation of the dimensions between state-owned and privately-owned companies.

A study of Chinese companies including construction companies by Luo and Liu [[25]] shows that the managers of the companies themselves also influence reporting, with companies having professionally networked managers showing better reporting quality. In addition, Zhang et al. [[87]] found in their study of the top five construction companies in China that internal factors such as rewards and corporate culture, among other factors, can also influence reporting on CSR activities and sustainability.

##### 4.2.2.2 Foci of content of sustainability reports

Overall, sustainability reports set rather different thematic priorities. Jones et al. [[70]] recognize a strong focus on environmental issues as well as on aspects relating to the workplace, the marketplace, and the community. Similar results were obtained from an analysis of CSR reports from international contractors, where a strong focus on community involvement and community development in the construction industry in the regions of Asia, EU, USA/Canada, and China was observed [[51]]. Lu et al. [[10]] identified content related to emissions, effluents, waste, and occupational health and safety as the most frequently reported content in the reports. In contrast, less information is reported on more sensitive topics such as anti-corruption, anti-competitive behavior, and intra-corporate information transparency [[10,88]]. Ye et al. [[59]] mention that CSR issues should be categorized into global and local issues. According to this view, companies should align their focus with global, universal issues as well as with local conditions, such as social measures related to the workplace conditions of employees.

In Malaysia, according to Siew's [[89]] study of property and construction companies, there is a transparent presentation of governance issues, but there is a lack of transparency concerning economic and social issues, which makes it difficult to compare the sustainability performance of companies. Many authors [[15,90,29,91]] confirm that the social dimension of sustainability, in particular, is neglected in the sustainability reports, while there is still a strong focus on constant growth and consumption and, therefore, on economic issues [[67,27,92,82,91,14]].

Saenz and Brown [[88]], on the other hand, report that the sustainability reports of construction companies in Latin America report on interactive training and the code of conduct for employees, and that the topics of control and evaluation are given a lot of space in the reports. Whang and Kim [[14]] note that social factors came to the fore; nevertheless, many measures are only used selectively, which is contrary to a necessary holistic view of SD [[81,58,14]].

Many studies [[68,81,80,76,85,87,93]] have found a focus on ecological topics in construction companies’ sustainability reports. A study of the Australian construction and real estate industry by Ang and Wilkinson [[28]] found that many managers and engineers believe that sustainability primarily brings environmental benefits, followed by economic and social ones.

Evangelinos et al. [[8]] note that construction companies in the UK do not report aspects of negative CSR performance, such as clarifying statements on sanctions and penalties that were imposed due to improper behavior. The choice of words in the reports is also selected very carefully, indicating that attempts are made to create a positive impression [[24]].

Considering the content of the individual dimensions in more detail, and in addition to the content mentioned above, topics in the ecological dimension deal with house greening, environmental agency partnerships, and environmental conservation organizations [[70]]. Often, there is also a focus on reporting on emissions, effluents, and waste [[26,94,10,95]], promoting engagement in CSR and adjusting policies to respond to environmental problems [[3,78]], governmental supervision and promotion of environmental protection activities [[78]], energy-related issues like energy efficiency, greenhouse gas emission reduction, and integration of renewable energy [[84]], environmental management capability development [[96]], sustainable and efficient resource and material use [[97,95]], or reducing the environmental impact in general [[98,95]]. Xie et al. [[17]] mention that ecological measures are taken into account and reported in the construction process but are no longer considered after the construction project has been completed.

The topics that were reported in the economic area include sourcing of goods/services, sales to customers, advice a company provides to clients, development of relationships with clients and suppliers, development of sustainability standards and procedures, benchmarking, and portfolio risk analysis, sustainability audits, socio-economic impact assessments, ethical investments, long-term sustainable growth models [[70]], market presence, global logistics, supply chain of construction business resources [[10]], bidding processes [[88]], competitive strength [[71]], steady business growth, company reputation, competitive advantage in international markets [[95]], profit-making, dividends paid to shareholders [[67]], and economic development [[73]].

Social aspects that are mentioned and presented in the sustainability reports of construction companies include commitment to employees, caring for staff for continuing success, remunerations, rewards, working conditions, well-being, diversity, health and safety, sustainability education [[15,70,10,29,58]], internal communication, interactions with communities [[27,3,70,58]], charitable donations, education, leadership [[70,88]], the built environment [[70]], labor practices [[78,51]], affordability [[98]], fair and equitable job opportunities, official labor contracts, participation in company decision-making processes [[51]], work-life balance, upholding the merit of “hard working” in the value system [[10]], gender policies, women on board [[89]], quality and safety [[27,3]], clients [[27,3]], CSR administration [[3]], anti-corruption [[27,88]], fair competition [[27]], human resource development and training [[67,29]].

##### 4.2.2.3 Sustainability reporting guidelines and audits

For the preparation of sustainability reports, assistance in the form of guidelines or standards is often used, e.g., the GRI reporting guidelines, which are intended to promote the credibility, transparency, and reliability of the reported information and to counteract the so-called “greenwashing” aspect. Many authors [[10,85,87]] found that a very large number of international construction companies use reporting guidelines (e.g., GRI, ISO26000) and frameworks (e.g., CASS-CSR) when preparing sustainability reports, which results in reports being easier to compare. Furthermore, many international construction companies are listed on the GRI website or the Dow Jones Sustainability Index [[84]]. Some international construction companies also use third-party auditors to review their reports and thus confirm the credibility, transparency, and reliability of the reported content [[82,10]].

However, there are studies that find that many companies still make insufficient use of frameworks and/or third-party assurance processes [[8,89]]. On the other hand, Isaksson and Steimle [[99]] emphasize that the GRI guidelines, for example, are not sufficient to clearly present SR. Glass [[41]] also notes that, although these standards help companies with their SR processes, they still come under criticism from time to time.

In conclusion, variations in the sustainability reports can be attributed to different aspects such as the geographical location of the companies, the development level of a country, or company size. In addition, the studies suggest that environmental and economic issues are more prominent in reporting than social issues. For the preparation of the reports, the companies use guidelines, frameworks, and audits, which are meant to help design and verify the reports as credible, transparent, and trustworthy.

The following chapter 4.2.3. serves to answer research question 3, where the embedding of SR in the CS management of construction companies is addressed.

#### 4.2.3 Sustainability reporting and corporate sustainability management

##### 4.2.3.1 Managing sustainability holistically

Sustainability requires a holistic view of the scope of all three dimensions - ecology, economy, and social affairs - which sometimes leads to difficulties for companies in balancing the three dimensions. Campos et al. [[83]] believe that a holistic implementation of sustainability is crucial for successful business development in the construction industry, but regional and local conditions must also be considered. Many companies in the construction industry still focus too much on single dimensions of sustainability [see e.g. Ref. [27]], and clear definitions of sustainability are lacking [[98]]. Activities or measures in these areas must therefore be viewed holistically. This begins with the consideration of these topics during strategy development and extends to implementation within the individual operational processes for project execution [[70,100]]. Arruda et al. [[67]] advise companies to push sustainability and incorporate it as a new element into management practices. In this context, Jones et al. [[82]] speak of a committed approach to materiality which is integrated into core business strategies to demonstrate the commitment to sustainability. In doing so, current economic, political, and institutional dynamics should be critically examined, and measures adapted to realize sincere efforts toward CS [[67]].

In addition, the time perspective of the company's goals plays an important role. Goals are often described as being more short-term oriented with a focus on economic benefits than long-term oriented through considering investments in sustainability measures [[14]]. Competitiveness and the associated financial advantages [[74]] could be strengthened in the long term through a focus on sustainability [[70,73,69]]. However, a long-term orientation is more susceptible to variations, for example with regard to the expectations of stakeholders, which is why it would be necessary to define widely accepted and required long-term goals [[49]]. Due to a focus on individual aspects and short-term goals, a large number of researchers [[92,22,101,91,102,14,103]] have called for a balanced consideration of the dimensions and involvement of stakeholders, which would in turn be useful for embedding, developing, and further shaping sustainability in the company and its environment including all stakeholders.

##### 4.2.3.2 Stakeholder management

Stakeholders and the market as a whole demand more reporting on sustainability, as awareness of sustainability is increasing. Involving stakeholders in the development of sustainable measures/ideas in a company can deliver successful results [[92,51,66,80,18,93]]. An increase in communication with stakeholders can also lead to the achievement of public value, improved marketing, public relations, and competitive advantages [[28,86]]. The interaction with stakeholders can also enrich the content of sustainability reports and thus, in turn, influence society's expectations of a company's sustainability measures [[22,10]]. Communication with stakeholders must, of course, be adapted and carried out depending on the proximity or affiliation to stakeholder groups: Abuzeinab and Arif [[47]] divide stakeholders into internal and external groups, with internal communication focusing on support, empowerment, incentives and rewards, collaborations, training, and effective communication, while the focus with external stakeholders is on the supply chains, customers, proactive communication, interaction and relationship building, incentive systems, partnerships, and the offering of different services [[47,70]]. As stakeholders are involved in various distinct phases of a construction project, a distinction can also be made between the project phase and organizational management [[50,100]].

In conclusion, the transparency and effectiveness of decisions should be presented and communicated to the stakeholders [[104]]. Bamgbade et al. [[75]] even speak of a complete people- and potentially customer-oriented execution of construction projects to be able to react quickly to competitive situations and remain innovative. However, it should also be considered that the approaches of stakeholders to the topic of sustainability can differ and thus trigger tensions that can influence the construction process. Changes in the consideration of sustainable measures lead to uncertainties and concerns of the stakeholders, therefore justifications are required for potential changes in sustainability management, as well as support and communication of largely accepted goals to the stakeholders [[49]].

In principle, there is an increasing trend in reporting on sustainability, which in turn means that construction companies recognize the relevance of reporting the company's commitments and achievements [[2,94,41,84]]. This trend is very often driven by the social pressure of the stakeholders, who want a transparent, open, and comparable presentation of a company's activities concerning sustainability [[41,46,82]]. Even companies that want to position themselves more internationally and grow can strengthen their public image through sustainable strategies [[96]]. Campos et al. [[83]] further argue that stakeholders should already be involved in the project development to achieve the most innovative manufacturing processes and supply chains.

##### 4.2.3.3 Instruments and concepts of sustainability management

Although SR, as described above, can be associated with many advantages for a company and its stakeholders, challenges can arise regarding the implementation or adaptation of sustainable measures and related reporting in a company. Such challenges can be triggered by the respective availability of technologies and resources to implement sustainable measures, a lack of incentive systems and advantages, pressure from stakeholders, loss of the company's image, personal attitudes, various regulations and standards, lack of support, low market acceptance, lack of proper performance measurements, lack of willingness and economic hurdles [[28,97,46,98,86,29,105]]. These challenges can be overcome/avoided through better management and governmental support in the implementation of sustainable measures, e.g., through knowledge-sharing, providing resources, the establishment of market competition mechanisms, and encouragement of firms for CSR practices. Furthermore, special CSR activities tailored to local regulations can help overcome hurdles in the implementation of sustainability measures, e.g., through a stronger focus on the needs of the respective stakeholders to set the necessary CSR measures in a specific region and situation to maintain legitimacy through corporate actions. Better organizational management with sustainability concepts and strategies, proper performance measurements, transparency in reporting processes, participation in training courses, research seminars, innovation projects, conferences, an exchange of experience within the construction industry, and employee training and education on sustainability could also help overcome these challenges and maintain or gain legitimacy [[96,18]].

In summary, sustainability must be implemented and further developed holistically in any given company, taking all dimensions into account and embedding it at the strategic level, to guarantee the company's success. This requires clear definitions, long-term goal orientation, and management practices which are currently lacking in many companies. In addition, the demand for information on sustainability measures from stakeholders is increasing, leading companies to communicate more intensively and to involve stakeholders in decisions and objectives according to their affiliation and proximity to a company. This in turn can help companies position themselves or become more innovative. Nevertheless, there are also some challenges associated with sustainability management in companies, such as the resources, regulations, and market mechanisms required for it. These challenges can be overcome through supportive government policies and better management practices.

## 5 Discussion of results

The status quo of the literature on SR in the construction industry shows that the companies in the industry have different motives for disclosure, that reporting has different focal points in terms of content and varying quality, and that this is caused by various influencing factors. In addition, it is shown what role reporting plays within CS management and that there are many opportunities and challenges. Nevertheless, it is also clear that the literature on SR in the construction industry is still at an early stage and important issues have not yet been sufficiently investigated or have been neglected altogether.

To answer why construction companies engage in SR (research question 1), the studies conclude that companies in the construction industry mainly pursue motives that are based either on instrumental or strategic as well as social/political considerations. Normative motives, such as the moral attitude of management or stewardship thinking, are hardly taken into account [e.g. Ref. [25]]. On the one hand, this suggests that companies in the construction industry are well aware of the effects their actions have on SD and also perceive pressure to justify themselves. Especially, because stakeholders are increasingly taking a critical look at the life cycles of buildings and infrastructure and the resulting impact on nature and society. On the other hand, they have also recognized that reporting beyond the financial bottom line can not only be used to meet societal expectations, satisfy stakeholders’ information needs, or legitimize entrepreneurial activity but also constitute an important instrument to control or enhance CS performance. Thereby, increased sustainability performance in turn can make a crucial or even vital contribution to the achievement of traditional corporate goals.

Although initial findings suggest a significant influence, the extent to which these motives depend on institutional framework conditions cannot be answered conclusively. In this context, for example, the economic development of a country [[10]] or compliance requirements from legal regulations, such as those resulting from Directive 2014/95/EU [[20]] and the CSRD [[21]], must be mentioned. Accordingly, in addition to analyzing normative motives, further research should also take a precise look at social/political motives to investigate the influence of institutional framework conditions more closely, especially against the background of rising societal expectations and increasing disclosure obligations.

Companies who have recognized the instrumental value of SR or have started reporting for this reason can fully exploit the potential if a holistic anchoring of the topic of sustainability is achieved and, in this sense, a link will be established with the control instruments or mechanisms already in place. Even if the motive for reporting is primarily based on the fulfillment of societal expectations or the adaptation to institutional requirements and the control potential is not to be used at all, it is essential that the disclosed information corresponds to the actual performances of the reported company. Otherwise, there is a risk that stakeholders will classify the reporting as greenwashing endangering the company's reputation as a result.

Regarding the design and content and, therefore, answering research question 2, the papers show variations with regard to the scope [e.g. Refs. [2,68]], the quality [e.g. Refs. [51,10]], or the focus [e.g. Refs. [70,90]]. The studies indicate that certain company-specific factors, such as the size of the company or the degree of internationalization, shape the design of reporting in the construction industry [[27,68,4,59,77,84]], as also found in other industries or cross-industry studies [e.g. Refs. [30,33]]. This influence can be justified from both an instrumental perspective and a social/political one. Larger and/or international companies are often more exposed to public awareness, which in turn increases the pressure to justify themselves. In addition, their more complex stakeholder structures often result in greater information asymmetries. Against this background, comprehensive and high-quality reporting has a considerable instrumental value, since it can be understood as a tried and tested means of reducing this information asymmetry and can therefore bring advantages.

The different foci in reporting can also be explained from both theoretical points of view. On the one hand, the strong focus on topics related to the ecological dimension might be a result of increased pressure to legitimize this area; on the other hand, it might also be the case that companies believe that this area is particularly relevant for them and that they, therefore, have a strong lever to contribute to SD. The same applies to the issue of occupational safety, which plays a more important role in the construction industry than in other industries. On the other hand, the omission of sensitive or negative aspects can be seen as the result of considerations of legitimation, especially if they are material. From this perspective, omitting certain topics might make sense, at least in the short term. In the medium to long term, however, there is a risk that stakeholders will doubt the credibility of the information disclosed. In contrast, orienting the reports to reporting guidelines, such as those of the GRI, or an audit can increase credibility or trustworthiness.

In this context, however, it must be mentioned that the literature to date still leaves many questions with regard to the aspect of materiality unanswered, although this topic has already received a lot of attention in the context of SR in other industries [e.g. Refs. [106,107]]. This is particularly remarkable considering that the construction industry has considerable specifics that suggest differences in terms of the materiality of various sustainability issues. Further studies should pay attention to this aspect of reporting and try to identify the specific issues that, among the multitude of sustainability topics, are particularly relevant to the industry. A conceivable material aspect here would be, for example, a stronger focus on reporting the long-term life cycles of buildings and infrastructure, and the associated measurement of the impact on nature and society. The findings can help both practitioners to identify the material topics, and standard-setters and legislators to develop or improve guidelines or disclosure obligations.

In 2015, the United Nations member states dedicated themselves to the 2030 Agenda in order to achieve SD. In doing so, 17 goals were formulated to foster SD [[108]]. Since then, companies across industries have also started to make reference to the SDGs in their sustainability reports. Against the background of materiality, some SDGs can be identified that are of particular relevance to the construction industry [[12,13]]. Assigning different reporting topics to the SDGs helps to present the contribution to the individual goals more transparently, which in turn can bring advantages for the reporting companies. For example, the reported content regarding occupational health and safety as well as work-life balance [e.g. Refs. [70,10]] for employees can be assigned to SDG 3 “Healthy Lives and Well-being”. SDG 4 “Quality Education” is also frequently cited indirectly in the literature through content such as interactive training for employees, continuing success of staff, human resource development and training, or participation in company decision-making processes [e.g. Refs. [67,51]]. Companies in the construction industry also cite content on gender policies and women on the board of directors in their reports [[89]], indicating consideration of SDG 5 “Gender Equality”. In the area of environmental topics, content on renewable energy, energy transition, or energy efficiency is considered [[84]], which can be assigned to SDG 7 “Affordable and Clean Energy”. Topics such as workplace, marketplace, anti-corruption, anti-competitive behavior, intra-corporate information transparency, benchmarking, working conditions, or leadership [e.g. Refs. [10,95]] belong to SDG 8 “Decent Work and Economic Growth”. SDG 9 “Industry, Innovation and Infrastructure” is also particularly relevant for the construction industry, and is addressed by the following topics mentioned in the literature: adjusting companies’ policies to respond to environmental problems, promoting engagement in CSR, development of relationships with and influence on suppliers and clients [e.g. Refs. [78,88]]. SDG 11 “Sustainable Cities and Communities” includes the topics of community development and house greening [[70,51]], and SDG 12 “Responsible Consumption and Production” mainly addresses environmental issues such as emissions, effluents, waste, sustainable and efficient resource and material use, sustainability standards and procedures [e.g. Refs. [26,95]]. SDG 13 “Climate Action” is addressed in general terms by reducing environmental impact [[98,95]], and SDG 17 “Partnerships for the Goals” mentions environmental agency partnerships and relationships with environmental conservation organizations [[70]].

Regardless of whether a company creates a link to specific SDGs or not, a balanced, transparent, and credible disclosure of information on SD is central so that reporting is not simply dismissed as a mere PR tool, or even worse, raises suspicions of greenwashing. However, this can only be one side of the coin for companies that want to improve their CS performance. Regarding the use of SR within CS management (research question 3), the literature suggests using the full potential that SR offers, meaning that the information that is collected and processed in the course of reporting should also be of use for CS management. Here, the literature already indicates different ways in which this can be done, or in other words, what possibilities exist for using the information for control purposes. Nevertheless, there is still a need for further research. The costs that arise from reporting are significant, so the information should not only be used for accountability purposes but also to manage or improve CS performance, as improved performance can bring many benefits.

## 6 Conclusion, limitations & implications

The overall consideration of the available literature on SR in the construction industry makes clear that, although comprehensive knowledge has already been gained, essential topics have still not been sufficiently researched. Hence, there is a need for further research. Our review aimed to present the status quo of the literature on SR in the construction industry. To this end, three research questions were posed, the first of which relates to the question of why construction companies engage in SR. The findings suggest that the drivers towards SR are often instrumental or social/political in nature. The second research question dealt with the contents and designs of the sustainability reports. The results showed strong variations in reporting, which can be traced back to a variety of factors. For reporting, companies often follow frameworks or standards and use audits to ensure transparency and reliability. Furthermore, the content of sustainability reports mentioned in the literature could be assigned to the different SDGs. The third research question dealt with how SR is embedded in CS management. The findings of the literature review have shown that sustainability must be anchored in the corporate strategy and corresponding measures must be implemented at the operational level for SD to be promoted holistically. This requires clear definitions and goals as well as an adaptation of management practices already in place.

Some limitations apply to this paper. Through a systematic literature review, we demonstrated the current state of SR in the construction industry. The creation of the search string, the selection of databases, the screening criteria, the assignment of information to thematic clusters, and the subsequent synthesis of the results can all be viewed as limitations even though the highest value was placed on transparency and comprehensibility in the literature search and analysis.

The main directions in which further research might go are related to the motives for reporting, in particular their contextualization and the additional consideration of normative motives, the identification or definition of the material topics for the industry, a stronger consideration of the longevity of buildings and infrastructure in reporting as well as considering the use of the information collected in the course of CS management.

By performing a systematic literature review in the field of SR in the construction industry, this study contributes to the academic debate in several ways. We were able to present the available knowledge considering the industry specifics for the construction industry, as it has been done in other industries [see, e.g., mining [32], aviation [33], oil and gas [109]]. In addition, we have presented different theoretical perspectives as explanatory approaches to the existing findings. These suggest that both the motivation and the designs and foci within reporting are particularly shaped by instrumental or social/political considerations. The theoretical perspectives also suggest that reporting information will not be used to the same extent for sustainability management in all companies.

Furthermore, this study has also practical implications. Companies in the construction industry must cover a wide range of information needs with their reporting, are confronted with reporting standards or obligations, and are increasingly expected to show their contributions to the United Nations' SDGs. The findings of our literature review can help companies create balanced reporting that considers industry requirements, which is particularly important to increase credibility and avoid greenwashing allegations. They also highlight the role reporting can play in sustainability management. In addition, the results can be used by standard setters and legislators to (further) develop requirements or standards for SR and thereby particularly address the specifics of the construction industry.

## Ethics statement

Review and/or approval by an ethics committee was not needed for this study because this study used a systematic literature review as a research method and therefore only used publicly available sources.

## Data availability statement

No data associated with this study has been deposited into a publicly available repository because the data is included in the references due to the article type of review.

## CRediT authorship contribution statement

**Daniela Cortés:** Conceptualization, Data curation, Formal analysis, Investigation, Methodology, Visualization, Writing – original draft, Writing – review & editing. **Albert Anton Traxler:** Conceptualization, Data curation, Formal analysis, Investigation, Methodology, Project administration, Writing – original draft, Writing – review & editing. **Dorothea Greiling:** Conceptualization, Formal analysis, Investigation, Methodology, Writing – review & editing.

## Declaration of competing interest

The authors declare that they have no known competing financial interests or personal relationships that could have appeared to influence the work reported in this paper.

## Footnotes

1 The search string was adapted to the respective requirements of the databases.

## References

[1] Kpmg International, Big Shifts, Small Steps: Survey of Sustainability Reporting 2022, 2022 Online: https://assets.kpmg/content/dam/kpmg/xx/pdf/2022/10/ssr-small-steps-big-shifts.pdf. (Accessed 2 November 2023).

[2] Afzal F., Lim B., Prasad D. (2017). An investigation of corporate approaches to sustainability in the construction industry. Procedia Eng..

[3] Jiang W., Wong J.K. (2016). Key activity areas of corporate social responsibility (CSR) in the construction industry: a study of China. J. Clean. Prod..

[4] Petera P., Wagner J., Pakšiová R., Křehnáčová A. (2019). Sustainability information in annual reports of companies domiciled in the Czech republic and the Slovak republic. Eng. Econ..

[5] Sev A. (2009). How can the construction industry contribute to sustainable development? A conceptual framework. Sustain. Dev..

[6] Spence R., Mulligan H. (1995). Sustainable development and the construction industry. Habitat Int..

[7] Xia B., Olanipekun A., Chen Q., Xie L., Liu Y. (2018). Conceptualising the state of the art of corporate social responsibility (CSR) in the construction industry and its nexus to sustainable development. J. Clean. Prod..

[8] Evangelinos K., Skouloudis A., Jones N., Isaac D., Sfakianaki E. (2016). Exploring the status of corporate social responsibility disclosure in the UK building and construction industry. Int. J. Global Environ. Issues.

[9] Fei W., Opoku A., Agyekum K., Oppon J.A., Ahmed V., Chen C., Lok K.L. (2021). The critical role of the construction industry in achieving the sustainable development goals (SDGs): delivering projects for the common good. Sustainability.

[10] Lu W., Ye M., Flanagan R., Ye K. (2016). Corporate social responsibility disclosures in international construction business: trends and prospects. J. Construct. Eng. Manag..

[11] Eurostat (2008). https://ec.europa.eu/eurostat/web/products-manuals-and-guidelines/-/ks-ra-07-015.

[12] Dar M.U.D., Shah A.I., Bhat S.A., Kumar R., Huisingh D., Kaur R. (2021). Blue Green infrastructure as a tool for sustainable urban development. J. Clean. Prod..

[13] Pandit A. (2017). Infrastructure ecology: an evolving paradigm for sustainable urban development. J. Clean. Prod..

[14] Whang S.W., Kim S. (2015). Balanced sustainable implementation in the construction industry: the perspective of Korean contractors. Energy Build..

[15] Evangelinos K., Fotiadis S., Skouloudis A., Khan N., Konstandakopoulou F., Nikolaou I., Lundy S. (2018). Occupational health and safety disclosures in sustainability reports: an overview of trends among corporate leaders. Corp. Soc. Responsib. Environ. Manag..

[16] Johnsson F., Karlsson I., Rootzén J., Ahlbäck A., Gustavsson M. (2020).

[17] Xie L., Xu T., Le Y., Chen Q., Xia B., Skitmore M. (2020). Understanding the CSR awareness of large construction enterprises in China. Adv. Civ. Eng..

[18] Zhang Q., Oo B.L., Lim B.T.H. (2019). Drivers, motivations, and barriers to the implementation of corporate social responsibility practices by construction enterprises: a review. J. Clean. Prod..

[19] Global Reporting Initiative (2022). https://www.globalreporting.org/how-to-use-the-gri-standards/gri-standards-english-language/.

[20] Directive 2014/95/EU of the European Parliament and of the Council of 22 October 2014 as regards disclosure of non-financial and diversity information Online: https://eur-lex.europa.eu/legal-content/DE/TXT/?uri=celex%3A32014L0095. (Accessed 2 November 2023).

[21] Directive 2022/2464/EU of the European Parliament and of the Council of 14 December 2022 as regards corporate sustainability reporting. https://eur-lex.europa.eu/eli/dir/2022/2464/oj.

[22] Le Roux C., Pretorius M. (2019). Exploring the nexus between integrated reporting and sustainability embeddedness. Sustainability Accounting, Management and Policy Journal.

[23] Traxler A.A., Schrack D., Greiling D. (2020). Sustainability reporting and management control–A systematic exploratory literature review. J. Clean. Prod..

[24] Harymawan I., Nasih M., Ratri M.C., Soeprajitno R.R.W.N., Shafie R. (2020). Sentiment analysis trend on sustainability reporting in Indonesia: evidence from construction industry. Journal of Security and Sustainability Issues.

[25] Luo J., Liu Q. (2020). Corporate social responsibility disclosure in China: do managerial professional connections and social attention matter?. Emerg. Mark. Rev..

[26] Chang A.S., Paramosa L.S., Tsai C.Y. (2021).

[27] Chang R.D., Zuo J., Zhao Z.Y., Soebarto V., Lu Y., Zillante G., Gan X.L. (2018). Sustainability attitude and performance of construction enterprises: a China study. J. Clean. Prod..

[28] Ang S.L., Wilkinson S.J. (2008). Is the Social Agenda Driving Sustainable Property Development in Melbourne Australia?. Property Manag.

[29] Mavroulidis M., Vouros P., Fotiadis S., Konstantakopoulou F., Fountoulakis G., Nikolaou I., Evangelinos K. (2022). Occupational health and safety of multinational construction companies through evaluation of corporate social responsibility reports. J. Saf. Res..

[30] Hahn R., Kühnen M. (2013). Determinants of sustainability reporting: a review of results, trends, theory, and opportunities in an expanding field of research. J. Clean. Prod..

[31] Kolk A. (2010). Trajectories of sustainability reporting by MNCs. J. World Bus..

[32] Lodhia S., Hess N. (2014). Sustainability accounting and reporting in the mining industry: current literature and directions for future research. J. Clean. Prod..

[33] Zieba M., Johansson E. (2022).

[34] Global reporting initiative (n.d.). Sector program. https://www.globalreporting.org/standards/sector-program/.

[35] Christensen H.B., Hail L., Leuz C. (2021). Mandatory CSR and sustainability reporting: economic analysis and literature review. Rev. Account. Stud..

[36] Minutiello V., Tettamanzi P. (2022). The quality of nonfinancial voluntary disclosure: a systematic literature network analysis on sustainability reporting and integrated reporting. Corp. Soc. Responsib. Environ. Manag..

[37] Schaltegger S., Jones S., Ratnatunga J. (2012). Contemporary Issues in Sustainability Accounting, Assurance and Reporting.

[38] Garcia-Torea N., Larrinaga C., Luque-Vílchez M. (2023). Bridging the understanding of sustainability accounting and organizational change. Organ. Environ..

[39] Cabeza L.F., Rincón L., Vilariño V., Pérez G., Castell A. (2014). Life cycle assessment (LCA) and life cycle energy analysis (LCEA) of buildings and the building sector: a review. Renew. Sustain. Energy Rev..

[40] Xiong B., Lu W., Skitmore M., Chau K.W., Ye M. (2016). Virtuous nexus between corporate social performance and financial performance: a study of construction enterprises in China. J. Clean. Prod..

[41] Glass J. (2012). The state of sustainability reporting in the construction sector. Smart and sustainable built environment.

[42] Chang R.D., Soebarto V., Zhao Z.Y., Zillante G. (2016). Facilitating the transition to sustainable construction: China's policies. J. Clean. Prod..

[43] Hansen E.G., Schaltegger S. (2016). The sustainability balanced scorecard: a systematic review of architectures. J. Bus. Ethics.

[44] Donaldson T., Preston L.E. (1995). The stakeholder theory of the corporation: concepts, evidence, and Implications. Acad. Manag. Rev..

[45] Jensen M.C., Meckling W.F. (1976). Theory of the firm: managerial behavior, agency costs, and ownership structure. J. Financ. Econ..

[46] Goel A., Ganesh L.S., Kaur A. (2019). Sustainability integration in the management of construction projects: a morphological analysis of over two decades' research literature. J. Clean. Prod..

[47] Abuzeinab A., Arif M. (2014). Stakeholder engagement: a green business model indicator. Procedia Econ. Finance.

[48] Comyns B., Figge F., Hahn T., Barkemeyer R. (2013). Sustainability reporting: the role of ‘search’, ‘experience’ and ‘credence’ information. Account. Forum.

[49] Herazo B., Lizarralde G. (2016). Understanding stakeholders' approaches to sustainability in building projects. Sustain. Cities Soc..

[50] Lin X., Ho C.M., Shen G.Q. (2017). Who should take the responsibility? Stakeholders' power over social responsibility issues in construction projects. J. Clean. Prod..

[51] Liao P.C., Xia N.N., Wu C.L., Zhang X.L., Yeh J.L. (2017). Communicating the corporate social responsibility (CSR) of international contractors: content analysis of CSR reporting. J. Clean. Prod..

[52] Neu D., Warsame H., Pedwell K. (1998). Managing public impressions: environmental disclosures in annual reports. Account. Org. Soc..

[53] Freeman R.E., Wicks A.C., Parmar B. (2004). Stakeholder theory and ‘the corporate objective revisited’. Organ. Sci..

[54] Suchman M.C. (1995). Managing legitimacy: strategic and institutional approaches. Acad. Manag. Rev..

[55] Brown N., Deegan C. (1998). The public disclosure of environmental performance information—a dual test of media agenda setting theory and legitimacy theory. Account. Bus. Res..

[56] Dowling J., Pfeffer J. (1975). Organizational legitimacy: social values and organizational behavior. Pac. Socio Rev..

[57] DiMaggio P.J., Powell W.W. (1983). The iron cage revisited: institutional isomorphism and collective rationality in organizational fields. Am. Socio. Rev..

[58] Passos Neto G., Kohlman Rabbani E.R., Valdes-Vasquez R., Alencar L.H. (2022). Implementation of the global reporting initiative social sustainability indicators: a multi-case study approach using Brazilian construction companies. Sustainability.

[59] Ye M., Lu W., Flanagan R., Chau K.W. (2020). Corporate social responsibility “glocalisation”: evidence from the international construction business. Corp. Soc. Responsib. Environ. Manag..

[60] Deegan C. (2014). An overview of legitimacy theory as applied within the social and environmental accounting literature. Sustainability accounting and accountability.

[61] Hasnas J. (1998). The normative theories of business ethics: a guide for the perplexed. Bus. Ethics Q..

[62] Sundin H., Granlund M., Brown D.A. (2010). Balancing multiple competing objectives with a balanced scorecard. Eur. Account. Rev..

[63] Fink A. (2019).

[64] Page M.J., McKenzie J.E., Bossuyt P.M., Boutron I., Hoffmann T.C., Mulrow C.D., Moher D. (2021). The PRISMA 2020 statement: an updated guideline for reporting systematic reviews. Int. J. Surg..

[65] Tranfield D., Denyer D., Smart P. (2003). Towards a methodology for developing evidence‐informed management knowledge by means of systematic review. Br. J. Manag..

[66] Lin X., McKenna B., Ho C.M., Shen G.Q. (2019). Stakeholders' influence strategies on social responsibility implementation in construction projects. J. Clean. Prod..

[67] Arruda L.R., de Jesus Lameira V., Quelhas O.L.G., Pereira F.N. (2013). Sustainability in the Brazilian heavy construction industry: an analysis of organizational practices. Sustainability.

[68] Huang C.F., Lu W.H., Lin T.T., Wu E.J. (2017). The current conditions of CSR implementation in construction industry: a lesson from Taiwan. Appl. Ecol. Environ. Res..

[69] Tan Y., Shen L., Yao H. (2011). Sustainable construction practice and contractors' competitiveness: a preliminary study. Habitat Int..

[70] Jones P., Roberts C., Hillier D., Comfort D., Clarke‐Hill C. (2009). Commercial property investment companies and corporate social responsibility. J. Property Invest. Finance.

[71] Chen P.H., Ong C.F., Hsu S.C. (2016). Understanding the relationships between environmental management practices and financial performances of multinational construction firms. J. Clean. Prod..

[72] Kresnanto N.C., Putri W.H. (2019). Lean and sustainable construction: link between the sustainability report disclosure and the impact on profitable opportunities for investors. IOP Conf. Ser. Mater. Sci. Eng..

[73] Liao P.C., Shih Y.N., Wu C.L., Zhang X.L., Wang Y. (2018). Does corporate social performance pay back quickly? A longitudinal content analysis on international contractors. J. Clean. Prod..

[74] Wang H., Lu W., Ye M., Chau K.W., Zhang X. (2016). The curvilinear relationship between corporate social performance and corporate financial performance: evidence from the international construction industry. J. Clean. Prod..

[75] Bamgbade J.A., Kamaruddeen A.M., Nawi M.N.M. (2017). Malaysian construction firms' social sustainability via organizational innovativeness and government support: the mediating role of market culture. J. Clean. Prod..

[76] Misopoulos F. (2021). Environmental and social sustainability in UK construction industry: a systematic literature review. European Journal of Economics and Business Studies.

[77] Ye M., Lu W., Xue F. (2022). Impact of institutional distance on environmental and social practices in host countries: evidence from international construction companies. J. Construct. Eng. Manag..

[78] Liao P.C., Liao J.Q., Wu G., Wu C.L., Zhang X.L., Ma M.C. (2018). Comparing international contractors' CSR communication patterns: a semantic analysis. J. Clean. Prod..

[79] Gou Z., Xie X. (2017). Evolving green building: triple bottom line or regenerative design?. J. Clean. Prod..

[80] Loosemore M., Lim B.T.H., Ling F.Y.Y., Zeng H.Y. (2018). A comparison of corporate social responsibility practices in the Singapore, Australia and New Zealand construction industries. J. Clean. Prod..

[81] Jones P., Comfort D., Hillier D. (2006). Corporate social responsibility and the UK construction industry. Economic research-Ekonomska istraživanja.

[82] Jones P., Hillier D., Comfort D. (2016). Materiality and external assurance in corporate sustainability reporting: an exploratory study of Europe's leading commercial property companies. Journal of European Real Estate Research.

[83] Campos J.K., Straube F., Wutke S., Cardoso P.A. (2017). Creating value by sustainable manufacturing and supply chain management practices–a cross-country comparison. Procedia Manuf..

[84] Zuo J., Zillante G., Wilson L., Davidson K., Pullen S. (2012). Sustainability policy of construction contractors: a review. Renew. Sustain. Energy Rev..

[85] Moon J., Parry T., Brown J. (2009). Corporate responsibility reporting in UK construction. Proceedings of the Institution of Civil Engineers-Engineering Sustainability.

[86] Lim B.T.H., Loosemore M. (2017). How socially responsible is construction business in Australia and New Zealand?. Procedia Eng..

[87] Zhang Q., Oo B.L., Lim B.T.H. (2022). Corporate social responsibility practices by leading construction firms in China: a case study. International Journal of Construction Management.

[88] Saenz C., Brown H. (2018). The disclosure of anticorruption aspects in companies of the construction sector: main companies worldwide and in Latin America. J. Clean. Prod..

[89] Siew R.Y.J. (2017). Critical evaluation of environmental, social and governance disclosures of Malaysian Property and Construction Companies. Construction Economics and Building.

[90] Lu Y., Zhang X. (2016). Corporate sustainability for architecture engineering and construction (AEC) organizations: framework, transition and implication strategies. Ecol. Indicat..

[91] Okanga B., Groenewald D. (2017). Leveraging effects of triple bottom lines business model on the building and construction small and medium-sized enterprises' market performance. Acta Commer..

[92] Illankoon I.C.S., Tam V.W., Le K.N., Shen L. (2017). Key credit criteria among international green building rating tools. J. Clean. Prod..

[93] Zuo J., Zhao Z.Y. (2014). Green building research–current status and future agenda: a review. Renew. Sustain. Energy Rev..

[94] Chang A.S., Romero A.M., Tsai C.Y. (2022). Environmental indicator disclosure of international contractors. J. Chin. Inst. Eng..

[95] Tan Y., Shuai C., Shen L., Hou L., Zhang G. (2020). A study of sustainable practices in the sustainability leadership of international contractors. Sustain. Dev..

[96] Chen P.H., Ong C.F., Hsu S.C. (2016). The linkages between internationalization and environmental strategies of multinational construction firms. J. Clean. Prod..

[97] Chang A.S., Canelas C., Chen Y.L. (2021). Relationships between environmental initiatives and impact reductions for construction companies. Sustainability.

[98] Isaksson R., Rosvall M. (2020).

[99] Isaksson R., Steimle U. (2009). What does GRI-reporting tell us about corporate sustainability?. The TQM Journal.

[100] Zhao Z.Y., Zhao X.J., Davidson K., Zuo J. (2012). A corporate social responsibility indicator system for construction enterprises. J. Clean. Prod..

[101] Manochin M.M., Jack L., Howell C. (2008). The boundaries of reporting sustainable development in social housing. Publ. Money Manag..

[102] Tetteh M.O., Chan A.P., Nani G. (2019). Combining process analysis method and four-pronged approach to integrate corporate sustainability metrics for assessing international construction joint ventures performance. J. Clean. Prod..

[103] Xing Y., Horner R.M.W., El-Haram M.A., Bebbington J. (2009). A framework model for assessing sustainability impacts of urban development. Account. Forum.

[104] Bassi A., Howard R., Geneletti D., Ferrari S. (2012). UK and Italian EIA systems: a comparative study on management practice and performance in the construction industry. Environ. Impact Assess..

[105] Yin B.C.L., Laing R., Leon M., Mabon L. (2018). An evaluation of sustainable construction perceptions and practices in Singapore. Sustain. Cities Soc..

[106] Jayarathna C.P., Agdas D., Dawes L., Miska M. (2021). Exploring sector-specific sustainability indicators: a content analysis of sustainability reports in the logistics sector. Eur. Bus. Rev..

[107] Slacik J., Greiling D. (2020). Coverage of G4-indicators in GRI-sustainability reports by electric utilities. J. Public Budg. Account. Financ. Manag..

[108] United Nations (n.d, Sustainable development goals Online: https://sdgs.un.org/goals. (Accessed 2 November 2023).

[109] Kwarto F., Nurafiah N., Suharman H., Dahlan M. (2022). The potential bias for sustainability reporting of global upstream oil and gas companies: a systematic literature review of the evidence. Management Review Quarterly.

